# Differences in Function and Healthcare Cost of Older Adults with Dementia by Long-Term Care Service Type: A National Dataset Analysis

**DOI:** 10.3390/healthcare9030307

**Published:** 2021-03-10

**Authors:** Ilsu Park, Kyounga Lee, Eunshil Yim, Kyunghee Noh

**Affiliations:** 1 Department of Healthcare Management, Dong-eui University, 176 Eomgwangno, Busanjin-gu, Busan 47340, Korea; ispark@deu.ac.kr; 2 Research Institute of Nursing Science, Seoul National University, 103 Daehak-ro, Jongno-gu, Seoul 03080, Korea; 3 Department of Nursing, Daegu Health College, 15 Yeongsong-ro, Buk-gu, Daegu 41453, Korea; yim7604@dhc.ac.kr; 4 Mokpo National Quarantine Station, 20 Haean-ro, 177 Beon-gil, Mokpo-si 58754, Korea; nohkh@korea.kr

**Keywords:** activities of daily living, cognitive function, medical cost, benefit-cost, long-term care, dementia

## Abstract

This study aims to analyze the function and cost changes among long-term care insurance (LTCI) beneficiaries with low-severity dementia according to their LTCI service type. Data were collected from the Korean LTCI and national health insurance (NHI) datasets. Participants were 4414 beneficiaries with dementia aged 65 or older who received LTC services continuously for 4 years (2008–2011). LTCI service types were classified into home care (HC), institutional care (IC), and combined care (CC). Activities of daily living (ADL), cognitive function, medical cost, and benefit-cost were assessed. Linear mixed models and multiple regression models were used to analyze the changes in function and costs of the beneficiaries. ADL, cognitive function, medical cost, and benefit-cost differed significantly depending on the service type and time (*p* < 0.001). LTCI service types affected the degree of changes in ADL, cognitive function, medical cost, and benefit-cost over four years and showed negative changes in IC and CC beneficiaries than HC beneficiaries. HC is a cost-effective way to maintain the function of beneficiaries with low-severity dementia. Thus, efforts are needed to actively promote HC services.

## 1. Introduction

About 50 million people worldwide experience dementia, with approximately 10 million new cases every year [1]. Dementia is a disease that impairs cognitive ability, disrupting daily functioning and requires constant symptom management and support of caregivers, especially in comparison to other diseases [2].

According to the Vienna International Plan of Action on Aging, the fundamental principle for older adults’ care is to ensure that they lead an independent life in the community for as long as possible. Dementia-friendly communities (DFCs) are an emerging organization working toward the development of a nationwide dementia management policy [3]. DFCs are UK-based policy initiatives aimed at ensuring that people with dementia are supported by and included in their local communities [4]. The number of patients with dementia in Korea is increasing rapidly, and 10.16% of the population aged 65 or older is reported to have dementia [5].

An increase in chronic diseases and population aging, a decrease in fertility rates, and changes in social and family norms have changed the care of the population of older persons from individual and family responsibilities to national responsibilities [6]. Since Korea is a single universal national health insurance (NHI) system, all people can receive medical service benefits from the government. However, in addition to medical services, benefits for physical assistance, household chores, and nursing care have become a national responsibility. Since July 2008, the Korean government has been providing substantial lifetime care support through institutional or home-based care services to those eligible for the long-term care insurance (LTCI) system.

All persons aged 65 and older may apply for LTCI benefits, as may persons younger than 65 years with the geriatric disease (e.g., dementia, Parkinson’s disease). To receive care services under the LTCI system, an applicant first requests an assessment of their care needs, which is conducted by the National Health Insurance Corporation (NHIC). NHIC staff visit the applicant at home and assess his or her care needs using the LTC approval checklist (LTCAC). The checklist includes functional conditions, environmental conditions, and need for benefit service in five areas (52 items), such as activities of daily living (ADL), cognitive function, behavior change, medical treatment, and rehabilitation needs [7,8]. The LTC approval score is generated on a 100-point scale by summing the scores in each of these five areas; the score determines the eligibility level. Applicants with a specific minimum score or higher are eligible for LTCI coverage [9]. The highest LTCI approval is level 1, which applies to beneficiaries who need help in all aspects of their daily life due to the poorest medical and functional status. In 2008, when the LTCI system was first started, only applicants with a score of 55 or higher became beneficiaries, but the minimum approval score gradually reduced from 2012. The eligibility level of the beneficiaries was defined via a three-level system in 2008, but this was changed to a five-level system in 2014. Therefore, the LTCI scheme gradually covered more beneficiaries.

LTCI primarily consists of two types of services: (1) Institutional care (IC) services aimed at providing beneficiaries with housing, meals, nursing care, and other conveniences necessary for daily functioning, and (2) home care (HC) services that enable beneficiaries to receive the necessary services from a professional caregiver while living in their own residence. Depending on the number of residents, IC facilities are divided into aged care facilities (10 or more persons) and elderly group homes (9 or fewer persons). HC services are divided according to the content of the services: Home visit care (home help and support with daily activities), home-visit nursing, home-visit bathing, day and night care, short-term respite care, and welfare equipment service [10].

LTCI is based on the principle of prioritizing HC services, but the use of IC services is increasing. In general, only level 1–2 beneficiaries who require extensive physical assistance and nursing care can use the IC services, but level 3 beneficiaries with dementia can also use IC services. The proportion of HC expenses gradually decreased from 56.7% in 2009 to 52.9% and 48.2% in 2011 and 2013, respectively, while the proportion of IC expenses increased from 43.3% in 2009 to 47.1% and 51.8% in 2011 and 2013, respectively [10]. In particular, IC service consumption is increasing among patients with dementia due to the burden on family members at home [11]. This is because when the beneficiaries’ health deteriorates, and they need more care, the option of using IC services rather than HC services is a more convenient choice, and thus, it is gradually becoming more popular. It has been reported that in cases where the family is responsible for providing care to the older adults with dementia, family members tend to experience the burdens of providing support, such as restrictions on social activities, negativity in their relationship with the older adults, psychological and financial strain, and physical health deterioration [12]. Caregiver burden is an important predictor of death or institutionalization of patients with dementia [13]. In high-income countries, it is common to admit people with dementia into institutions; 50% of patients with dementia are reported to live within such institutions [14].

It is difficult to determine which LTCI service type is more effective. There are mixed results in the health outcomes of IC and HC beneficiaries. A previous study reported that cognitive and physical functions improved more in HC groups, while behavioral symptoms improved in IC groups [8]. However, multiple issues have been reported with IC services: social isolation among patients, service quality, and lack of privacy due to multi-patient rooms within facilities. These infrastructural issues have led to various problems, such as difficulty in adapting to the new environment, deterioration in the quality of life, abuse, and so on; therefore, it is essential to support older adults with dementia at home for as long as possible [11]. It is important for people with dementia to be integrated into the community to the extent possible rather than placing them in institutions.

As stated above, LTCI beneficiaries in Korea were initially classified into three levels, where levels 1 and 2 included highly dependent beneficiaries, while level 3 contained the least dependent beneficiaries in terms of the need for daily life support. More than half of the beneficiaries belonged to level 3, which was the group with the lowest severity [15]. Levels 1 and 2 need full or substantial assistance in daily living, so they may require IC services; in contrast, level 3 beneficiaries should be focused on HC services. However, beneficiaries with dementia may be admitted to institutions to avoid the burden of providing care at home. Therefore, it is necessary to provide objective evidence by evaluating the effects of LTCI service types on both the beneficiaries’ function and their socioeconomic aspects. Depending on the type of LTCI service, there will be a change in beneficiaries’ ADL and cognitive functions and differences in medical and benefit-costs. Since LTCI only provides benefits for physical support, assistance for household tasks, and nursing care, medical services must be provided through NHI. Older persons need both healthcare and long-term care. More than 90% of LTCI beneficiaries have chronic illnesses, and most of these persons use medical services through NHI [15]. Therefore, it is necessary to analyze whether there is a difference between the cost of using LTCI benefits and the cost of using medical services according to LTCI service type.

Beneficiaries with dementia and their families should be provided with information according to the LTCI service type to enable them to make informed decisions; additionally, healthcare providers and policymakers should be aided in developing an effective LTCI system using these findings. Therefore, this study analyzes the difference in the effects of the functional and socioeconomic aspects of beneficiaries according to LTCI service type for level 3 beneficiaries with dementia. Specifically, the distribution of beneficiaries according to LTCI service type was characterized and a comparison made of ADL, cognitive function, medical costs, and benefit-costs. Changes were characterized in ADL, cognitive function, medical costs, and benefit-costs of beneficiaries from 2008 to 2011, and the impact analyzed of LTCI service types on these four-year changes.

## 2. Materials and Methods

### 2.1. Study Design

This study analyzed national-level LTCI data and NHI data received from the Korea NHIC. The NHIC is a national institution that is responsible for the management of NHI and LTCI. NHI supports various medical-related services (outpatient care, inpatient stay, pharmaceuticals, and selective Korean traditional medicines) for the entire population of Korea [16]. LTCI supports physical activities, household chores, and nursing needs for older persons with various difficulties in performing daily living activities due to geriatric disease or old age [9]. Participants were level 3 beneficiaries, aged 65 or older, with dementia in 2008, when the LTCI was introduced. Of these, data of those who continued to use LTCI up to 2011 were analyzed because the criteria for the classification of LTCI beneficiaries in Korea were changed in 2012. Beneficiaries were divided into three groups: (1) those receiving only IC, (2) those receiving only HC, and (3) those receiving combined care (CC), i.e., alternating between IC and HC. The ADL, cognitive function, medical cost, and benefit-cost of each group were compared, and the effect of LTCI service type on the function and cost of beneficiaries was analyzed. This study was approved by the institutional review board of the Catholic University of Korea (MIRB00602004).

### 2.2. Study Sample

**Figure 1:**
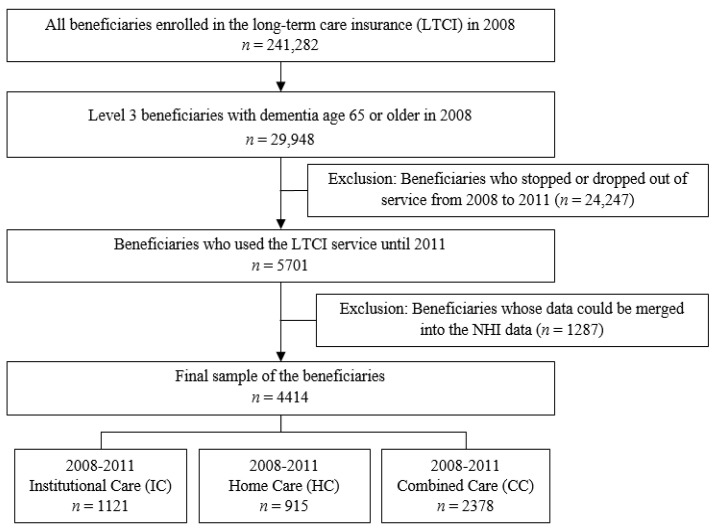
Study sample selection flow.

We identified participants from the LTCI data set based on the following criteria: (1) aged 65 or older, (2) with dementia, (3) classified as level 3, (4) continued to use LTCI from 2008 to 2011, and (5) whose data could be merged into the NHI data. In 2008, there were 29,948 beneficiaries (65 years and above) with dementia who belonged to the level 3 category of the LTCI. This accounted for 0.60% of the Korean population over 65 years old and 7.1% of the older adults with dementia in 2008 over 65 years of age. Of these, 5701 beneficiaries continued to use LTCI up to 2011. Since the medical cost had to be calculated, 1287 beneficiaries whose data could be merged into the NHI data were excluded. The final sample of this study was 4414 beneficiaries. Figure 1 shows the study sample selection flow over four years (2008–2011).

### 2.3. Study Measures

This study assessed general characteristics, type of LTCI services (IC, HC, and CC), ADL, cognitive function, medical cost, and benefit-cost of participants. General characteristics refer to participant characteristics such as sex, age, healthcare insurance type, living conditions (alone/with family), and primary caregiver prior to enrollment in LTCI. AS Korea has a universal NHI system, healthcare insurance type divided into NHI and Medical Aid (provided to low-income people). Primary caregiver refers to the participant’s primary caregiver(s) prior to the individual being included in the LTCI scheme. This includes spouses, children, and employed formal caregivers.

ADL and cognitive function scores were measured by LTCAC. LTCAC is a standardized instrument used to evaluate the care needs of beneficiaries [7,8]. The Korean government assesses all beneficiaries using this LTCAC, which determines the benefit eligibility level of beneficiaries. All beneficiaries are evaluated every 12 months, as mandated by the Korean government. ADL is an important aspect of maintaining physical function among older adults with dementia. It consists of 13 items: changing clothes, washing face, brushing teeth, bathing, eating, changing position, sitting up, transferring to a different seat, exiting a room, using the toilet, control of bowel movement, control of bladder, and washing hair. It uses a 3-point Likert-type scale ranging from 1 (totally independent) to 3 (totally dependent). The scores ranged between 13 and 39 points. Higher scores on the scale indicated higher physical functioning dependency of the participants. Cognitive function consists of 10 items: short-term memory loss, time disorientation, place disorientation, age disorientation (date of birth), inability to understand instructions, decreased situation judgment, communication problems, inability to calculate, inability to understand one’s daily schedule, and inability to recognize people. Each item was scored as 1 (existence of symptoms) or 0 (no symptoms). The total score ranges between 0 and 10 points. Higher scores on the scale indicate lower cognitive functioning of the participants.

Medical cost was calculated as the annual medical expenses of the beneficiaries for NHI services, while benefit-cost is the annual cost of using LTCI services. LTCI beneficiaries receive support for their physical activities, household chores, and nursing needs through LTCI. However, if a beneficiary becomes sick and needs medical services, such as hospitalization or medication, they receive medical services through NHI. As individuals enter the long-term care system, the need for disease management of these beneficiaries does not disappear [13]. Therefore, in this study, medical cost refers to the annual cost of receiving medical services through NHI, and benefit-cost refers to the annual cost of receiving benefits through LTCI. However, since Korea’s LTCI was introduced in July 2008, the benefit-cost in 2008 reflects only the six-month cost from July to December.

### 2.4. Data Analysis

The LTCI and NHI data were merged to calculate the medical cost of the LTCI beneficiaries. Chi-squared tests and analysis of variance (ANOVA) were performed to determine the differences in demographic characteristics, ADL, cognitive function, medical cost, and benefit-cost according to LTCI service type. Compared to 2008 ADL and cognitive function, if ADL and cognitive function scores increased in 2009, 2010, and 2011, they were classified as having deteriorated, while persons with decreased scores were classified as having improved. We analyzed a fixed effect of service type and random effects of time and interaction between time and service type, using a linear mixed model (LMM). Lastly, multiple regression analyses were performed to evaluate the association between service type and 4-year changes in ADL, cognitive function, medical cost, and benefit-cost. Covariates included in the multiple regression models were demographic information (sex, age), health insurance type, primary caregiver prior to enrollment in LTCI, and baseline ADL and cognitive function scores. Data were analyzed using SAS 9.2 (SAS Inc., Cary, NC, USA).

## 3. Results

### 3.1. Baseline Characteristics of Participants

Table 1 shows the general characteristics, ADL, cognitive function, medical cost, and benefit-cost of participants according to the type of LTCI services (IC, HC, and CC). Of the 4414 beneficiaries, 1121 were eligible for IC, 915 for HC, and 2378 for CC, i.e., those alternating between IC and HC. Over 80% of the beneficiaries were female. The average age of IC beneficiaries was the highest (IC 80.4 ± 7.1 years, HC 78.9 ± 6.9 years, and CC 79.4 ± 6.7 years). While there were many NHI beneficiaries for insurance types, in the case of IC, the proportion of medical aid was as high as 59.9%. It was found that 6.8% and 11.3% of IC and HC beneficiaries, respectively, lived alone. Additionally, 97% of IC beneficiaries had formal caregivers prior to enrolling in the LTCI, while 54.9% of HC beneficiaries had their children as their primary caregivers. IC beneficiaries scored higher than the overall average for ADL and cognitive function, while HC and CC beneficiaries scored lower than the overall average. Furthermore, CC beneficiaries incurred the highest annual medical costs, followed by HC and IC beneficiaries. Additionally, IC beneficiaries incurred the highest benefit-costs, which were 2.5 times those of HC beneficiaries.

### 3.2. Deterioration, Maintenance, and Improvement of ADL and Cognitive Function

Table 2 shows the deterioration proportions of ADL and cognitive function over time among the beneficiaries. Over time, beneficiaries’ ADL and cognitive function deteriorated. After 1 year, the deterioration proportions of ADL and cognitive functions were 59.3% and 38.5%, respectively, but after 3 years, these values increased to 78.1% and 52.5%, respectively. Deterioration of ADL was higher than that of cognitive function.

ADL and cognitive deterioration proportions differed by LTCI service type (*p* < 0.001). The CC group exhibited the greatest deterioration in ADL and cognitive function. Com-paring IC and HC groups, after 3 years, ADL had deteriorated to a greater extent in the IC group (78.6%) than in the HC group (71.0%). However, regarding cognitive function, deterioration was higher in the HC group (50.7%) than in the IC group (45.5%). The maintained and improved proportions were highest in the HC group for ADL (7.4% and 21.5%) and highest in the IC group for cognitive function (22.5% and 32.0%). In the CC group, both ADL and cognitive function had the lowest maintained and improved proportions.

### 3.3. ADL, Cognitive Function, Medical Cost, and Benefit-Cost in LTCI Beneficiaries Based on Service Type and Time

Table 3 shows the changes in ADL, cognitive function, medical cost, and benefit-cost, according to the service type and time. The results showed statistically significant differences according to service type and time. IC beneficiaries had the highest scores for ADL and cognitive function as per service type, and the scores increased over time. The annual medical cost incurred was lowest in the IC group and highest in the HC group according to service type (F = 26.78, *p* < 0.001). Additionally, cost decreased in 2009 and 2010, but significantly increased in 2011 (F = 14.77, *p* < 0.001). In contrast, benefit-costs incurred were highest in the IC group and lowest in the HC group (F = 2125.38, *p* < 0.001), and showed a significant increased over time (F = 11,878.17, *p* < 0.001).

### 3.4. Factors Influencing the 4-Year Changes in LTCI Beneficiaries

Table 4 shows the results of multiple regression analyses of the differences between ADL, cognitive function, medical cost, and benefit-cost in 2008 and 2011 as dependent variables; all the models were significant (*p* < 0.001). After adjusting for covariates, the LTCI service type was analyzed with respect to the amount of change in ADL, cognitive function, medical cost, and benefit-cost of LTCI beneficiaries. The changes in ADL, cognitive function, medical cost, and benefit-cost of the IC group were significantly higher than those of the HC group. With respect to the CC group, change in ADL, cognitive function, and benefit-cost were higher, while the change in medical cost was lower than that of the HC group.

In addition to LTCI service type, sex, caregiver, ADL score at baseline, and cognitive function score at baseline were associated with changes in ADL and cognitive function. Medical cost was associated with age, insurance type, caregiver, and ADL score at baseline, while age and caregiver were associated with the change in benefit-cost (*p* < 0.001).

## 4. Discussion

This study explored the ADL, cognitive function, medical cost, and benefit-costs according to LTCI service type (IC, HC, CC) for LTCI beneficiaries with dementia aged 65 or older. Older adults with dementia require considerable care, which incurs high caregiver burden and costs [17,18]. Therefore, it is necessary to develop national-level interventions to manage older adults with dementia. The goal of dementia care is to care for patients with dementia by integrating them into the community to the extent possible. However, many patients reside in institutions where they can receive dementia care. The Korean government provides national-level care for older adults with dementia within the LTCI system, divided into IC and HC. It is necessary to analyze the effect of service type to provide scientific evidence to the families and policymakers regarding appropriate care. The results of our study can be discussed with respect to the following three aspects: characteristics of LTCI beneficiaries by service type, the degree of deterioration of function according to service type, and the effect of the service type on changes in function and costs associated with the beneficiaries.

In this study, more beneficiaries were receiving IC than HC services. This is consistent with previous findings that the use of IC is more common [10,11]. In the case of IC, beneficiaries can receive services for 24 h, but in the case of HC, beneficiaries can only receive services for a limited time during the day, which increases the burden of care for dementia beneficiaries [10]. Therefore, beneficiaries and their families tend to use IC more than HC services. Since this is against the nationwide dementia management policy (community-based care) [3], it is necessary to consider ways to extend HC service hours and expand services. In terms of the beneficiary’s characteristics, we found that beneficiaries receiving IC services were older than those receiving other service types. Additionally, there were more people with low-income using Medical Aid in the IC group, and the ADL and cognitive functions of the IC group were worse than those of the HC group. The HC beneficiaries had relatively better ADL and cognitive function. The LTCI system is designed to use HC services first [19]. Therefore, IC services should be used when the health condition is more severe, and it is difficult to obtain an adequate residence due to low economic status. Level 3 beneficiaries are relatively less dependent among LTCI beneficiaries; thus, there are such persons who live alone and take care of themselves without any other caregivers before entering the LTCI system. Older adults who live alone have a high risk of depression, social isolation, poor quality of life, and health problems [20]. This emphasized the need to provide appropriate care to beneficiaries through the LTCI system. In addition, before entering the LTCI system, more than 80% of HC beneficiaries’ primary caregivers were family (spouse, children), but more than 90% of IC beneficiaries’ primary caregivers were formal caregivers. The families of HC beneficiaries (spouse, children) were able to provide care as primary caregivers. In contrast, IC beneficiaries were unable to receive care from their families; thus, most were in the care of formal caregivers or already resided in private facilities to support their care needs. In this respect, the distribution of beneficiaries according to IC and HC types was appropriate. IC benefits were being used by beneficiaries who had difficulty receiving care within their families, were of lower economic status, and had worse physical and cognitive function. Most previous studies conducted in other countries have also shown that the older persons with the most disabilities are more likely to receive IC services than HC services [21].

Dementia is a progressive, age-related, and chronic disease [22]; as such, its management is critical to limit deterioration. Regarding the change in functional aspects of the beneficiaries, the deterioration of ADL and cognitive function differed by LTCI service type. Deterioration of ADL was greater in the IC group than the HC group but higher in the HC group for cognitive function deterioration. This is consistent with previous studies showing mixed results in health outcomes for IC and HC recipients. Some studies have reported that beneficiaries’ physical/cognitive function deteriorated when using IC [8,23], while other studies reported that HC beneficiaries’ symptoms worsened [24,25]. These mixed results may be due to not taking into account differences in the beneficiaries’ baseline functional state, background, or other factors such as pharmacological services. However, deterioration of ADL and cognitive function was greater in the CC group than in the IC and HC groups. This result is consistent with a prior study of LTCI beneficiaries with high dependence and dementia [8]. This indicates that changes in the environment negatively affected the functioning of older people with dementia. Whether transferring from HC to IC or vice versa, changes in the environment will affect beneficiaries. Furthermore, multiple regression analysis after adjusting for covariates revealed that the fewest negative changes in ADL and cognitive function over four years occurred in the HC group. This can be interpreted as showing that HC, which was associated with the least change in the environment, is helpful for maintaining the ADL and functional status of older persons with dementia. Several studies have shown that changes in the environment can have negative physical and psychological effects on older adults, especially those with dementia or weakness [8,26,27]. Care management strategies for people with dementia need to identify which environmental factors negatively stimulate and increase stress and intervene before these factors disturbing to them. Noise and crowding can cause behavioral symptoms in people with dementia if they live in institutions such as nursing homes [28]. In addition, IC services can have a negative impact on beneficiaries due to the lack of personnel to provide adequate treatment at the institution, social isolation, maladjustment to new environments, and poor quality of life [11,29]. Therefore, older adults with dementia should remain in their community to the extent possible to avoid environmental pressures caused by the institutional admission. It is necessary to expand the LTCI service types from institution-oriented services to home- and community-based services, consistent with global trends [30].

This study also revealed that ADL, cognitive function, medical cost, and benefit-cost differed by time and service type. ADL and cognitive function among beneficiaries with dementia deteriorated over time, which in turn led to an increase in medical and benefit-costs. From a socioeconomic perspective, the HC group had the lowest total cost (combined medical and benefit-cost), consistent with earlier findings that reported HC could improve beneficiaries’ function at a lower cost [19]. Since HC beneficiaries lived at home, they could visit hospitals and clinics more frequently, even for small medical needs and symptoms, compared to IC beneficiaries. This may have led to increased medical costs for HC beneficiaries. However, the benefit-cost of HC beneficiaries was significantly lower than that of the other groups. The total combined medical and benefit-costs were the lowest in the HC group. This is similar to previous analyses of healthcare and LTC expenditures of community-living and institutionalized patients with dementia. Community-based dementia care reduces overall costs from a payer’s perspective, even if healthcare cost is high because the LTC costs are significantly lower [31]. As about 60% of the world’s population with dementia is living in low- and middle-income countries, cost-effective dementia management strategies are necessary [1]. Home-based services have proved to be more cost-effective than institution-based services to manage dementia. However, if LTC services are provided on a home and community basis, a formal and professional caregiver is essential. This is supported by the finding that the 4-year changes in ADL, cognitive function, medical cost, and benefit-cost were significantly higher when the former primary caregiver was an informal caregiver, such as a spouse or child, versus a formal caregiver. In particular, when the primary caregiver was a spouse, changes in ADL, cognitive function, and medical cost were greatly affected. This demonstrates that when a spouse, who is also aging, is the primary care provider, this has a negative effect on beneficiaries’ health and socioeconomic aspects. This reflects the high undermet care needs of the oldest old people when their older children over 60 are caregivers [32]. This is consistent with previous results showing that cognitive decline and disability progression could be reduced if older adults with dementia lived in the community and received care from a formal caregiver using a daycare center or various services for a certain amount of time a day. It is important to provide an appropriate level of care and support for people with dementia to help them to function effectively and be included within the community [7].

The findings above suggest that HC services should be actively promoted when caring for old adults with dementia, particularly for those with low severity. It also suggests that providing care within the home and community is more socioeconomically effective than institutional care. HC services can aid beneficiaries to avoid a socially isolated life and restore mental and physical functions so that they can live independently. In addition, it would be possible to mediate family crises by reducing the care burden or relieving the desire to place the older person in an institution so that beneficiaries can live in an integrated way within the community.

A limitation of this study is the lack of description of the various sub-services available under IC and HC service types. IC facilities are divided into aged care facilities and elderly group homes according to the size of the facility (number of beneficiaries residing in the facility). HC is divided into home visit care, home-visit nursing, home-visit bathing, day and night care, and short-term respite care, according to the service content. According to these sub-service types, differences in physical or cognitive function of beneficiaries and medical or benefit expenses will occur. However, we did not differentiate among these detailed service types. Second, we could only use the limited variables included in the LTCI dataset. Variables not included in our study (e.g., education level, location of residence, other healthcare services or treatments used by the beneficiaries, psychotropic medication, and so on) also affect beneficiaries’ physical and cognitive functions. Therefore, for a more detailed analysis, differences between beneficiaries should be analyzed using variables not included in the LTCI dataset. In addition, there were differences in baseline characteristics among the three different LTCI service types in this study. Although we adjusted for these baseline characteristics as a covariate, an analysis method that minimizes selection bias, such as propensity score matching, should be used in future studies. Nevertheless, this study is meaningful in that it presented differences in functional and socioeconomic aspects according to LTCI service type for LTCI beneficiaries with low severity dementia. In addition, since this study showed changes in beneficiaries over the past four years, this is a basis for suggesting which LTCI services should be further developed.

## 5. Conclusions

As the number of old adults with dementia increases, so does the burden of caring for them. Responsibility for caring for older adults with dementia is shifting from an individual to a national basis. In Korea, the government provides care for older adults with dementia within the LTCI system. Therefore, it is necessary to provide evidence that the government can use to determine how to effectively provide care for such persons. In this study, differences in ADL, cognitive function, and costs associated with old adults with low severity dementia were analyzed according to LTCI service type. ADL, cognitive function, medical cost, and benefit-cost differed by LTCI service type. In addition, regarding changes in health status and cost aspects of older adults with dementia, HC services had less influence on negative changes than did IC services after adjusting for covariates. For dementia management, it is important to delay the progression of deterioration in condition and function. The results of this study imply that HC can cost-effectively delay the decline in ADL and cognitive function of older adults with dementia. Therefore, various policies and awareness programs are needed to promote the use of HC services for older adults with low-severity dementia. These current data have significant implications for caring for the old adults with dementia that will be of use to policymakers, caregivers, dementia patients, and their families as the global demand for dementia care increases. However, further ongoing research is needed, given that the results of this study may have been affected by differences in the participant’s baseline characteristics and other uncontrolled factors.

## Figures and Tables

**Table 1:** Baseline characteristics of beneficiaries with dementia according to long-term care insurance (LTCI) service type.

Characteristic		Total	Institutional Care	Home Care	Combined Care	*p*-Value
*N*		4414	1121	915	2378	−
Sex *n* (%)	Female,	3735 (84.6)	971 (86.6)	733 (80.1)	2031 (85.4)	<0.001
	Male	679 (15.4)	150 (13.4)	182 (19.9)	347 (14.6)	
Age	mean ± SD	79.6 ± 6.9	80.4 ± 7.1	78.9 ± 6.9	79.4 ± 6.7	<0.001
	65–74, *n* (%)	1116 (25.3)	251 (22.4)	254 (27.8)	611 (25.7)	<0.001
	75–84, *n* (%)	2125 (48.1)	524 (46.7)	441 (48.2)	1160 (48.8)	
	≥85, *n* (%)	1173 (26.6)	346 (30.9)	220 (24.0)	607 (25.5)	
Insurance type *n* (%)	Medical Aid	1352 (30.6)	671 (59.9)	216 (23.6)	465 (19.6)	<0.001
	NHI	3062 (69.4)	450 (40.1)	699 (76.4)	1913 (80.4)	
Alone, *n* (%)		427 (9.7)	76 (6.8)	103 (11.3)	248 (10.4)	<0.001
Primary caregiver, *n* (%)	None	154 (3.5)	3 (0.3)	49 (5.4)	102 (4.3)	<0.001
	Spouse	604 (13.7)	1 (0.1)	247 (27.0)	356 (15.0)	
	Children	1860 (42.1)	30 (2.7)	502 (54.9)	1328 (55.8)	
	Formal	1796 (40.7)	1087 (97.0)	117 (12.8)	592 (24.9)	
ADL score (mean ± SD)		21.3 ± 3.2	22.1 ± 3.6	21.3 ± 3.1	20.8 ± 3.0	<0.001
Cognitive function score (mean ± SD)		6.6 ± 2.0	6.8 ± 2.0	6.1 ± 2.0	6.4 ± 1.9	<0.001
Medical cost (USD) mean ± SD		2452 ± 3322	2362 ± 2405	2913 ± 3351	2997 ± 3991	<0.001
Benefit-cost (USD) mean ± SD		2989 ± 2382	5565 ± 1234	2208 ± 1160	2076 ± 1814	<0.001

LTC = long-term care, NHI = national health insurance, SD = standard deviation, ADL = activities of daily living.

**Table 2:** Deterioration, maintenance, and improvement of ADL and cognitive function over time according to service type (*n*,%).

Variable		Total	Institutional Care	Home Care	Combined Care	*p*-Value
ADL	2008 to 2009					
	Deteriorated	2618 (59.3)	686 (61.2)	480 (52.5)	1452 (61.1)	<0.001
	Maintained	700 (15.9)	162 (14.5)	166 (18.1)	372 (15.2)	
	Improved	1096 (24.8)	273 (24.4)	269 (29.4)	554 (23.3)	
	2008 to 2010					
	Deteriorated	3100 (70.2)	835 (74.5)	570 (62.3)	1695 (71.3)	<0.001
	Maintained	399 (9.0)	81 (7.2)	91 (9.9)	227 (9.5)	
	Improved	915 (20.7)	205 (18.3)	254 (27.8)	456 (19.2)	
	2008 to 2011					
	Deteriorated	3449 (78.1)	881 (78.6)	650 (71.0)	1918 (80.7)	<0.001
	Maintained	249 (5.6)	52 (4.6)	68 (7.4)	129 (5.4)	
	Improved	716 (16.2)	188 (16.8)	197 (21.5)	331 (13.9)	
Cognitive function	2008 to 2009					
	Deteriorated	1700 (38.5)	399 (35.6)	323 (35.3)	978 (41.1)	<0.001
	Maintained	1210 (27.4)	303 (27.0)	260 (28.4)	647 (27.2)	
	Improved	1504 (34.1)	419 (37.4)	332 (36.3)	753 (31.7)	
	2008 to 2010					
	Deteriorated	2079 (47.1)	486 (43.4)	406 (44.4)	1187 (49.9)	<0.001
	Maintained	944 (21.4)	246 (21.9)	200 (21.9)	498 (20.9)	
	Improved	1391 (31.5)	389 (34.7)	309 (33.8)	693 (29.1)	
	2008 to 2011					
	Deteriorated	2319 (52.5)	510 (45.5)	464 (50.7)	1345 (56.6)	<0.001
	Maintained	839 (19.0)	252 (22.5)	173 (18.9)	414 (17.4)	
	Improved	1256 (28.5)	359 (32.0)	278 (30.4)	619 (26.0)	

ADL = activities of daily living.

**Table 3:** ADL, cognitive function, medical cost, and benefit-cost in LTCI beneficiaries according to service type and time.

Variable		Service Type (Mean ± SD)			F (*p*-Value)		
		Institutional Care	Home Care	Combined Care	Service Type	Time	Time by Service Type
ADL	2008	22.2 ± 3.6	21.4 ± 3.2	20.9 ± 3.0	78.45 (<0.001)	1519.61 (<0.001)	21.95 (<0.001)
	2009	24.8 ± 5.2	22.8 ± 4.5	23.3 ± 4.8			
	2010	27.1 ± 5.9	24.0 ± 5.5	25.2 ± 5.7			
	2011	28.6 ± 6.6	26.3 ± 6.7	27.7 ± 6.5			
Cognitive function	2008	6.8 ± 2.0	6.2 ± 2.1	6.5 ± 1.9	40.48) (<0.001)	259.63 (<0.001)	16.58 (<0.001)
	2009	6.8 ± 2.0	6.1 ± 2.1	6.7 ± 2.1			
	2010	7.0 ± 2.1	6.4 ± 2.1	7.0 ± 2.0			
	2011	7.2 ± 2.1	6.7 ± 2.2	7.3 ± 2.1			
Medical cost (USD)	2008	2362 ± 2405	2913 ± 3351	2997 ± 3991	26.78 (<0.001)	14.77 (<0.001)	6.43 (<0.001)
	2009	2213 ± 2397	2772 ± 3443	2759 ± 3628			
	2010	2148 ± 2322	2852 ± 3783	2640 ± 3419			
	2011	2478 ± 3328	3651 ± 5494	2781 ± 3989			
Benefit-cost (USD)	2008	5565 ± 1234	2208 ± 1160	2076 ± 1814	2125.38 (<0.001)	11,878.17 (<0.001)	517.41 (<0.001)
	2009	12,414 ± 2044	7190 ± 1943	8192 ± 3271			
	2010	13,486 ± 1900	7962 ± 1883	11,481 ± 3161			
	2011	13,302 ± 2570	7703 ± 2263	12,683 ± 2990			

LTCI = long-term care insurance, SD = standard deviation, ADL = activities of daily living.

**Table 4:** Multiple regression analysis of the effect of LTC on 4-year changes in ADL, cognitive function, medical cost, and benefit-cost.

		4-Year Changes (2011–2008)							
		ADL		Cognitive Function		Medical Cost		Benefit-Cost	
		Estimate (SE)	*p*	Estimate (SE)	*p*	Estimate (SE)	*p*	Estimate (SE)	*p*
Intercept		11.864	<0.001	3.22	<0.001	−579.70	0.605	5052.87	<0.001
LTC type (ref. home care)	Institutional care	3.29 (0.21)	<0.001	0.49 (0.09)	<0.001	1269.78 (0.11)	<0.001	2940.39 (0.35)	<0.001
	Combined care	1.87 (0.14)	<0.001	0.50 (0.11)	<0.001	−648.57 (−0.06)	0.001	5198.26 (0.70)	<0.001
Sex (ref. male)	Female	0.98 (0.05)	<0.001	0.33 (0.05)	<0.001	−399.68 (−0.03)	0.069	182.96 (0.02)	0.173
Age		0.01 (0.01)	0.388	0.02 (0.05)	<0.001	28.97 (0.04)	0.010	−14.30 (−0.03)	0.036
Insurance type (ref. NHI)	Medical Aid	0.20 (0.01)	0.583	0.19 (0.04)	0.087	−691.62 (−0.06)	0.015	240.27 (0.03)	0.165
Caregiver (ref. formal caregiver)	None	0.75 (0.02)	0.188	0.13 (0.01)	0.477	1794.17 (0.07)	<0.001	989.37 (0.05)	<0.001
	Spouse	2.74 (0.14)	<0.001	0.39 (0.06)	0.001	2887.29 (0.20)	<0.001	871.24 (0.08)	<0.001
	Children	1.21 (0.09)	<0.001	0.10 (0.02)	0.271	2281.76 (0.22)	<0.001	683.98 (0.09)	<0.001
ADL score at baseline		−0.61 (−0.29)	<0.001	−0.06 (−0.08)	<0.001	−107.05 (−0.07)	<0.001	3.30 (0.01)	0.659
Cognitive function score at baseline		0.27 (0.08)	<0.001	−0.60 (−0.52)	<0.001	54.17 (0.02)	0.153	44.74 (0.02)	0.054
Adj R^2^		0.112		0.280		0.041		0.325	
F (*P*)		51.76 (<0.001)		156.76 (<0.001)		18.24 (<0.001)		194.10 (<0.001)	

LTC = long-term care, SE = standard error, NHI = national health insurance, ADL = activities of daily living.
